# The Temperamental Factor in Disease

**Published:** 1908-07-04

**Authors:** 


### The Hospital

A Journal of

The Medical Sciences and Hospital Administration.

New Series. No. 69, Vol. III. [No. 1141, Vol. XLIV.] SATURDAY, JULY 4, 1908.

### The Temperamental Factor in Disease.

We have on a previous occasion commented upon the tendency, almost inevitable in schools of medicine, to focus attention upon the material side of bodily disorders, to the neglect of the moral and mental side and to the great loss both of the patient and of those receiving instruction. This misfortune is inherent in our present hospital-teaching for a variety of reasons. In the first place, the call upon the in-patient accommodation precludes the admission of any but those whose organic disorder is so grave as to monopolise attention; for when a man is in the throes of pneumonia or typhoid fever, or is half unconscious through cerebral thrombosis or Bright's disease, there is no temptation to pause and consider his state of mind. In the out-patient rooms, on the other hand, where one might expect to find the milder disorders, into whose clinical picture temperamental considerations are known to enter, many disabilities combine to accentuate the physical and discount the psychical aspects of the problems afforded. This is partly due to the exigencies of examinations. Wherever selection is practised in the *clientèle* sent for treatment to an out-patient department, the choice of the selector falls naturally upon such people as illustrate those definite organic disorders, compatible with tolerable activity, which are likely to meet the examinee when he submits himself for qualification. In such circumstances the student's mental horizon is prone to be bounded by what may be called the set pieces of out-patient practice; phthisis, to wit, and organic valvular disease of the heart, diabetes and chronic nephritis, cirrhosis of the liver, emphysema, osteo-arthritis, and a variety of chronic organic nervous lesions. The net result of such a training, is that, for the student, nosology is almost equivalent to the study of grave organic lesions. It is not until he enters upon private practice that he finds, to his astonishment, that the bulk of the people to whom he has to minister present a symptom-complex which is remarkable for the absence of physical signs of disease. Slight headaches, made prominent by their persistence rather than their severity, vague complaints of lassitude, lack of appetite, "indigestion," troubled sleep and depression of spirits, with a host of similar symptoms which his trafficking with urgent diseases has shadowed with neglect, now become a conspicuous ingredient of his mental preoccupations. He cannot afford to neglect them, and finds with regret and surprise that they are not so readily dismissed by gentian, rhubarb, and nux vomica, as his previous experience has led him to believe. If he is gifted with sensibility, or can command the advice of an experienced physician, he proceeds to work out his own salvation, realising at last that the symptoms which he has treated with so much assiduity and so little success are, indeed, only symptoms. That the headache and lassitude are the bodily expressions of *res angusta domi* or financial anxieties in general; that the "indigestion" is largely mental, functional perhaps to begin with, but confirmed and rendered chronic by an abiding recollection that a father or a brother died of gastric cancer, and by a fear that such a fate is again impending: that the insomnia is but the offspring of alarm, based upon the widespread belief that a few nights of sleeplessness are a source of danger to the reason, and self-perpetuating through the fears it arouses. Thus gradually he forms the conclusion that the armament of a physician is not complete when he has mastered the *materia medica*, the detection of an aortic murmur, or the features which distinguish syringo-myelia from progressive muscular atrophy; that the interactions of mental and bodily influences have to be taken very seriously into consideration if he is to deal with minor maladies to his patient's advantage and his own credit. Thus at long last, and not without grumbles at the default of his medical school, which has sent him out so ill-equipped in an item of his trade for which the call is perpetual, he sets himself to the study of temperament, and, ceasing to concern himself so much with the vagaries of erring viscera, devotes himself to the management of the complex personalities which possess them.

We have seen that the source of his grumbles is in part the demand for such studies as lead to success in examination. But this is not the sole obstacle to a more efficient schooling in this particular branch of medicine. There is another, both more potent and less easily obviated, namely, the publicity of the out-patient room. Although it is true that there are some people, sufferers from the class of maladies which is under question, who do not allow considerations of publicity to inhibit their descriptions of what they suffer, it is none the less certain that a great majority are more than commonly sensitive. It is idle to expect from such persons a public confession of the private anxieties, or pathogenic auto-suggestions, which so often underlie the manifestations of which they complain. Sometimes it is possible to detect outward signs of emotion which give the key to the temperament at work, but a neurotic constitution is not seldom combined with a power of self-control in public, which baffles all attempts to penetrate the surface until privacy has allayed the instinct of repression. This need for the establishment of confidential relations between the physician and his patient—the matter is of far less moment to the surgeon, though even he cannot afford to disregard it—will be obvious to any thoughtful person. Yet our present system shows little inclination to meet it. We believe that Déjerine makes a practice of seeing in privacy such of the cases attending his clinic as appear to be in need of exceptional treatment, and the departure seems so obviously reasonable, as to commend itself without further argument. Certainly to listen to a catalogue of miseries is more laborious than to prescribe a stomachic, but neither callousness or idleness can be charged against the out-patient service of our hospitals. What appears to be required is a little more of the imaginative faculty in those who conduct it; a little more interest in the estimation of temperament and the part it plays in the manifestations of many disorders, trivial in themselves, but often inexpressibly distressing to their victims.

